# Combined Cholecystoenteric Fistula and Choledocholithiasis: A Report of a Rare Case

**DOI:** 10.7759/cureus.76608

**Published:** 2024-12-30

**Authors:** Mila Kovacheva-Slavova, Victor Dimitrov, Plamen Gecov, Branimir Golemanov, Borislav Vladimirov

**Affiliations:** 1 Gastroenterology, University Hospital Tsaritsa Ioanna, Medical University of Sofia, Sofia, BGR; 2 Medical Imaging, University Hospital Tsaritsa Ioanna, Medical University of Sofia, Sofia, BGR

**Keywords:** cholecystoenteric fistula, choledocholithiasis, chronic calculous cholecystitis, endoscopic therapy, impaired liver function

## Abstract

Cholecystoenteric fistulas are a rare complication of chronic gallstone disease. If not diagnosed on time, they can cause several complications such as gallstone ileus, gastric outlet obstruction (Bouveret syndrome), cholangitis, or liver abscess. We present a case of a patient with chronic calculous cholecystitis, who was admitted due to unspecific abdominal discomfort and impaired liver function with increased cholestatic liver enzymes. We found a stone in the distal part of the common bile duct with the papilla orifice located in a duodenal diverticulum and a cholecystoduodenal fistula as an additional finding, which at that point was uncomplicated. After successful endoscopic therapy of the choledocholithiasis, the patient was referred for laparoscopic cholecystectomy and treatment of the fistula. During follow-up, the patient was in an excellent overall condition.

## Introduction

Cholecystoenteric fistulas are a rare complication of chronic gallstone disease and occur in 0.1-0.5% of patients with cholelithiasis, more often diagnosed in older female patients [1]. Spontaneous tracts between the gallbladder and the digestive tract might also be caused by duodenal ulcers perforation into the biliary tree, a neoplastic infiltration, Caroli’s disease, liver or renal abscesses, penetrating trauma, or colitis [2]. The altered physiology, which lacks protective mechanisms, can cause bile reflux into the duodenum or enteric content into the biliary tract [3]. Cholecystoduodenal fistula is the most common type of cholecystoenteric fistulas, found in 60-85% of patients, followed by cholecystocolic and cholecystogastric fistulas [4]. Reported complications of cholecystoenteric fistulas are gallstone ileus, gastric outlet obstruction (Bouveret syndrome) with or without acute pancreatitis, suppurative cholangitis, or liver abscess [1]. We present a rare case of a male patient who was admitted due to impaired liver function and was diagnosed with chronic cholecystitis and choledocholithiasis, duodenal diverticulum, and a cholecystoduodenal fistula as an additional finding. We excluded other causes for pneumobilia such as a biliary-enteric surgical anastomosis and an incompetent sphincter of Oddi. The clinical presentation was unspecific with moderate epigastric discomfort as the patient was diagnosed before any severe complications. We performed endoscopic extraction of several stones from the common bile duct, as the papilla orifice was located in a large periampullary duodenal diverticulum. After the successful endoscopic procedure, the patient underwent a laparoscopic deconnection of the biliodigestive fistula and cholecystectomy.

## Case presentation

**Figure 1:**
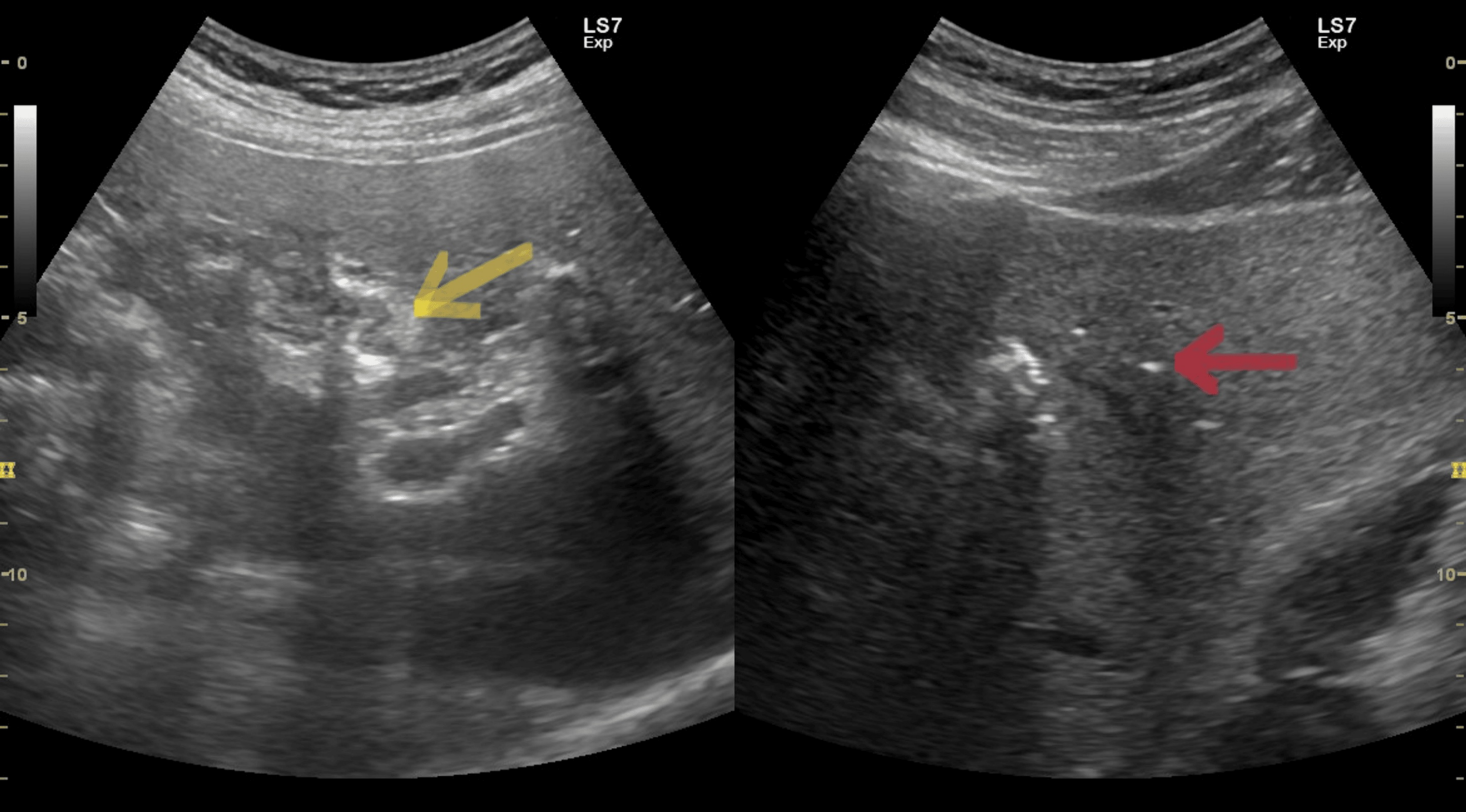
Suspected biliodigestive fistula (yellow arrow) and aerobilia (red arrow).

A 49-year-old man was admitted to the gastroenterology department due to liver dysfunction and moderate epigastric discomfort for a few weeks after eating fatty food. He reported several past episodes of biliary colic and chronic gallstone disease without any other comorbidities. At admission, he was afebrile. The complete blood count and coagulation status were within reference ranges, however, ALT (alanine aminotransferase) was 296 U/l (normal levels below 40 U/l), AST (aspartate aminotransferase) was 74 U/l (normal levels below 40 U/l), alkaline phosphatase was 325 U/l (normal levels below 150 U/l), GGT (gamma-glutamyl transpeptidase) was 1984 U/l (normal levels below 50 U/l). Bilirubin and amylase levels were normal at 12.1 umol/l and 27 U/l, respectively. We found dyslipidemia with total cholesterol 7.43 mmol/l (normal levels below 5.2 mmol/l), triglycerides 2.56 mmol/l (normal levels below 1.7 mmol/l), LDL (low-density lipoprotein) cholesterol 4.64 mmol/l (normal levels below 3.4 mmol/l) and HDL (high-density lipoprotein) cholesterol 1.32 mmol/l (normal above 1.45 mmol/l). C-reactive protein was slightly elevated (1.51 mg/dl by normal levels below 0.6 mg/dl). Markers for hepatitis B and C were negative. We performed an abdominal ultrasound and found a slightly enlarged liver with homogeneous hypoechoic echotexture. The gallbladder was minimally distended with double-contoured walls as in chronic calculous cholecystitis. The bile ducts were not dilated but with the presence of relatively mild aerobilia and some reflection of the walls of the bile ducts as in cholangitis. We suspected a spontaneous cholecystoenteric fistula (Figure 1).

**Figure 2:**
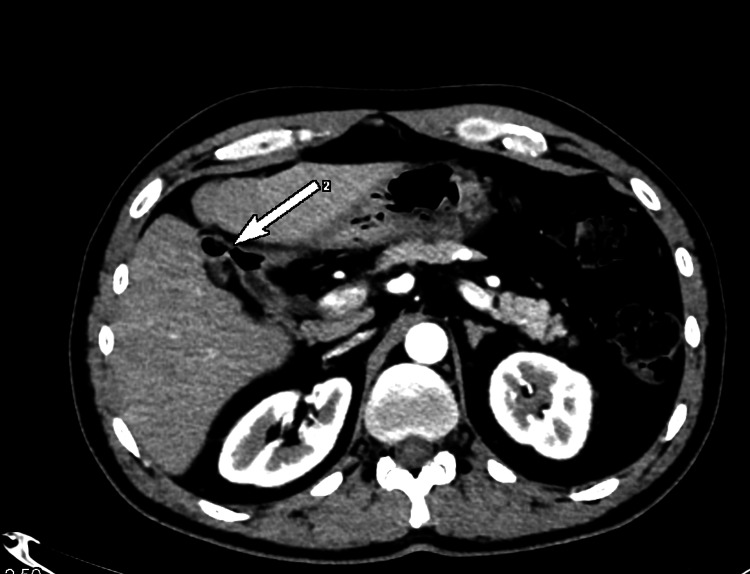
Computed tomography, axial view; a fistula between the corpus of the gallbladder and the bulb of the duodenum (white arrow).

**Figure 3:**
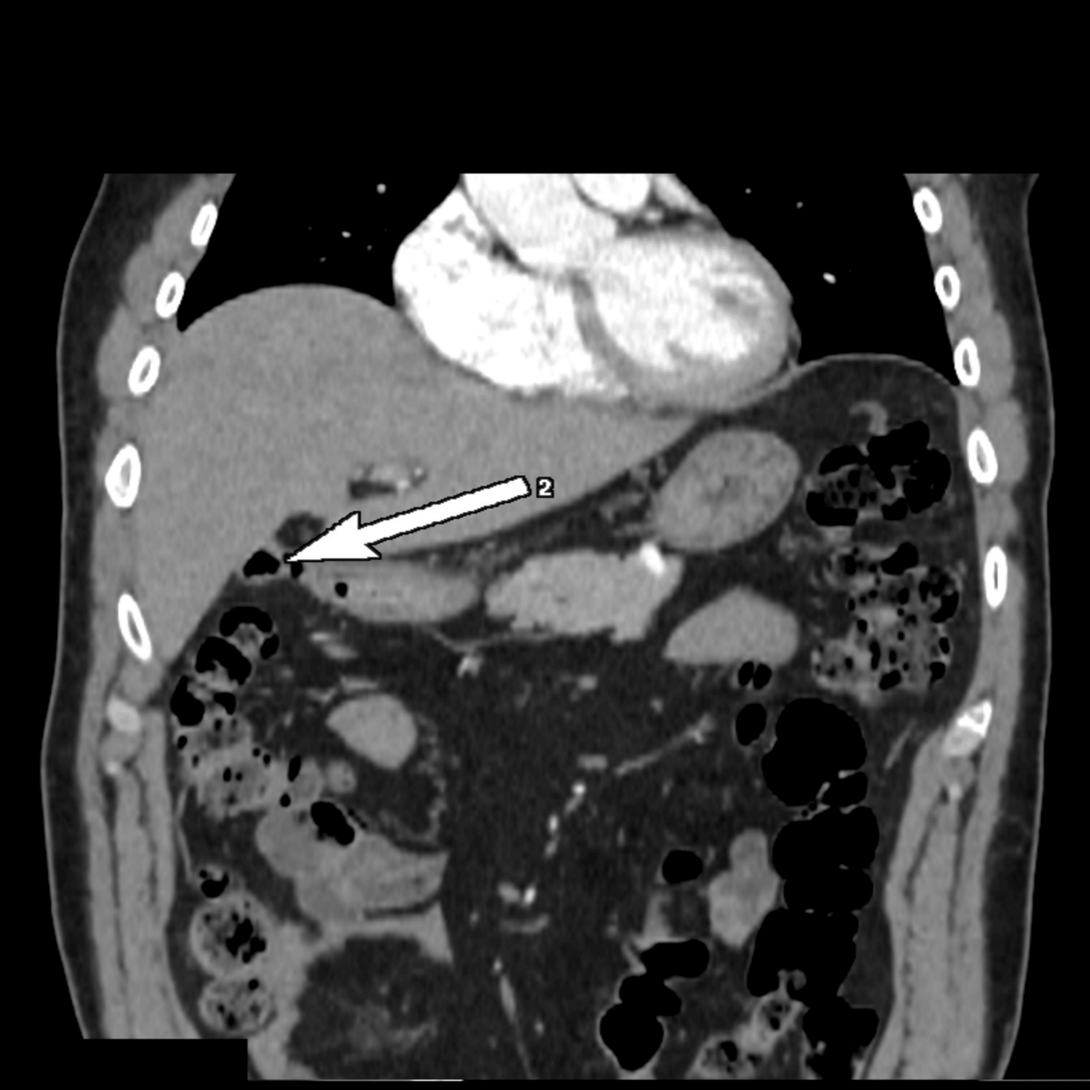
Computed tomography, coronary view; a fistula between the corpus of the gallbladder and the bulb of the duodenum (white arrow).

The size of the common bile duct distally was measured up to 10 mm. We further performed a computed tomography scan and found a fistula between the corpus of the gallbladder and the bulb of the duodenum (Figures 2, 3).

**Figure 4:**
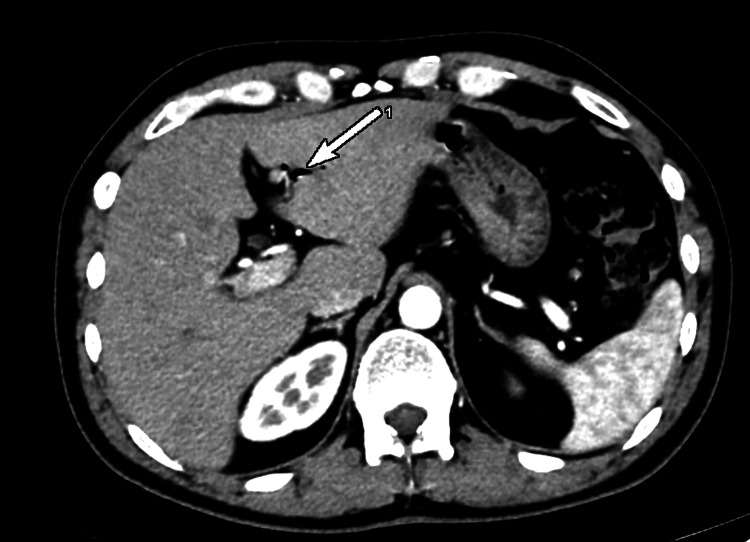
Computed tomography; intrahepatic aerobilia (white arrow).

There was an intrahepatic aerobilia (Figure 4).

**Figure 5:**
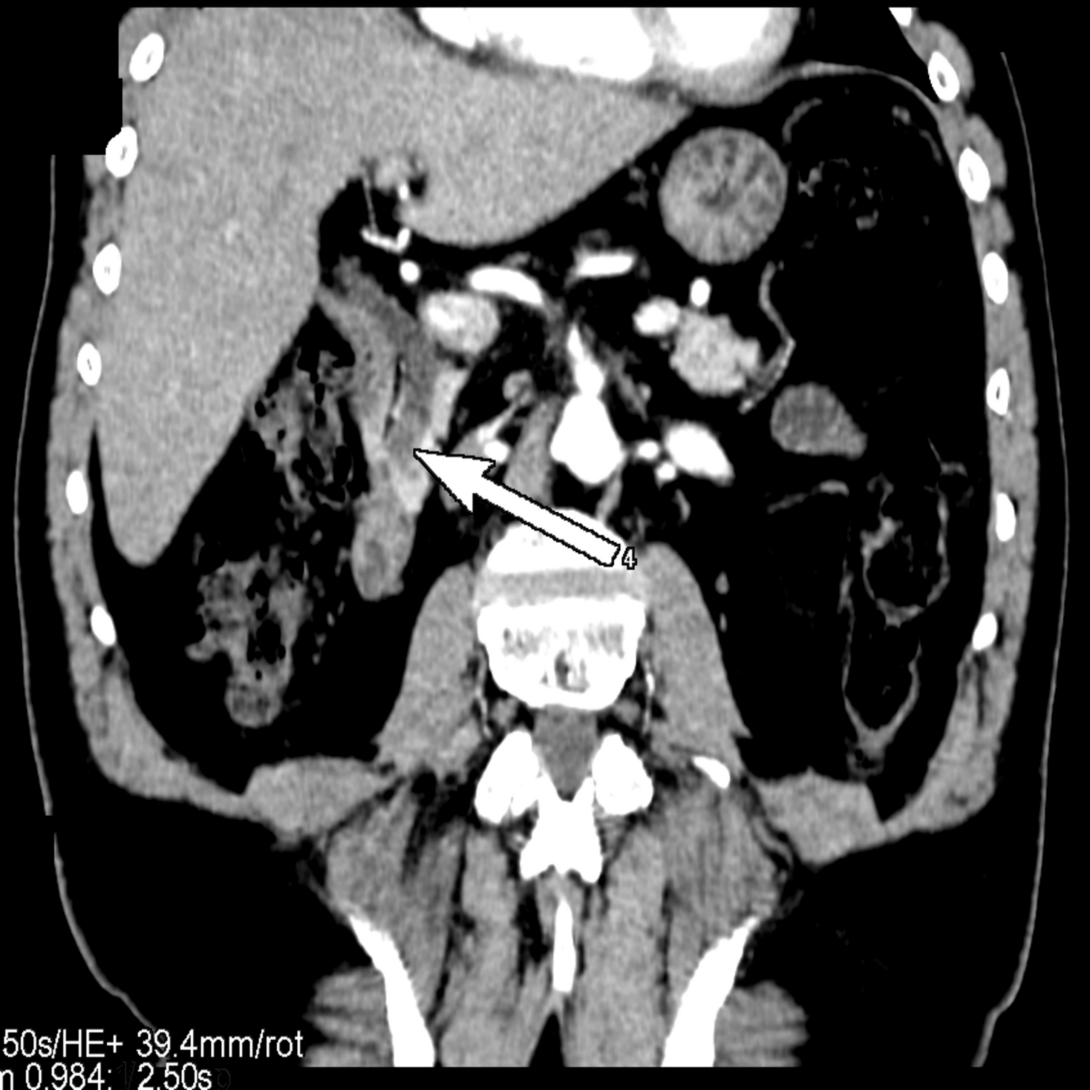
Computed tomography, coronary view; dilated extrahepatic bile duct (white arrow).

**Figure 6:**
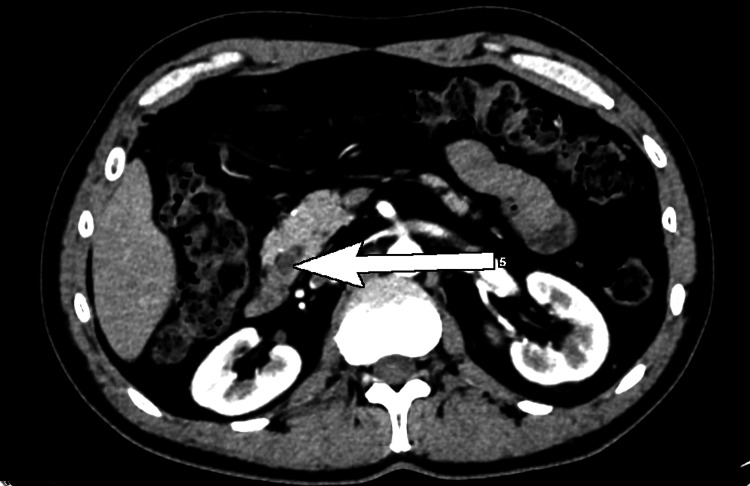
Computed tomography, axial view; a concrement with a diameter of 8.5 mm (white arrow) in the intrapancreatic part of the common bile duct.

Moreover, we diagnosed choledocholithiasis. The common bile duct was 9.6 mm wide and in its intrapancreatic part, we observed a stone with a diameter of 8.5 mm (Figures 5, 6).

**Figure 7:**
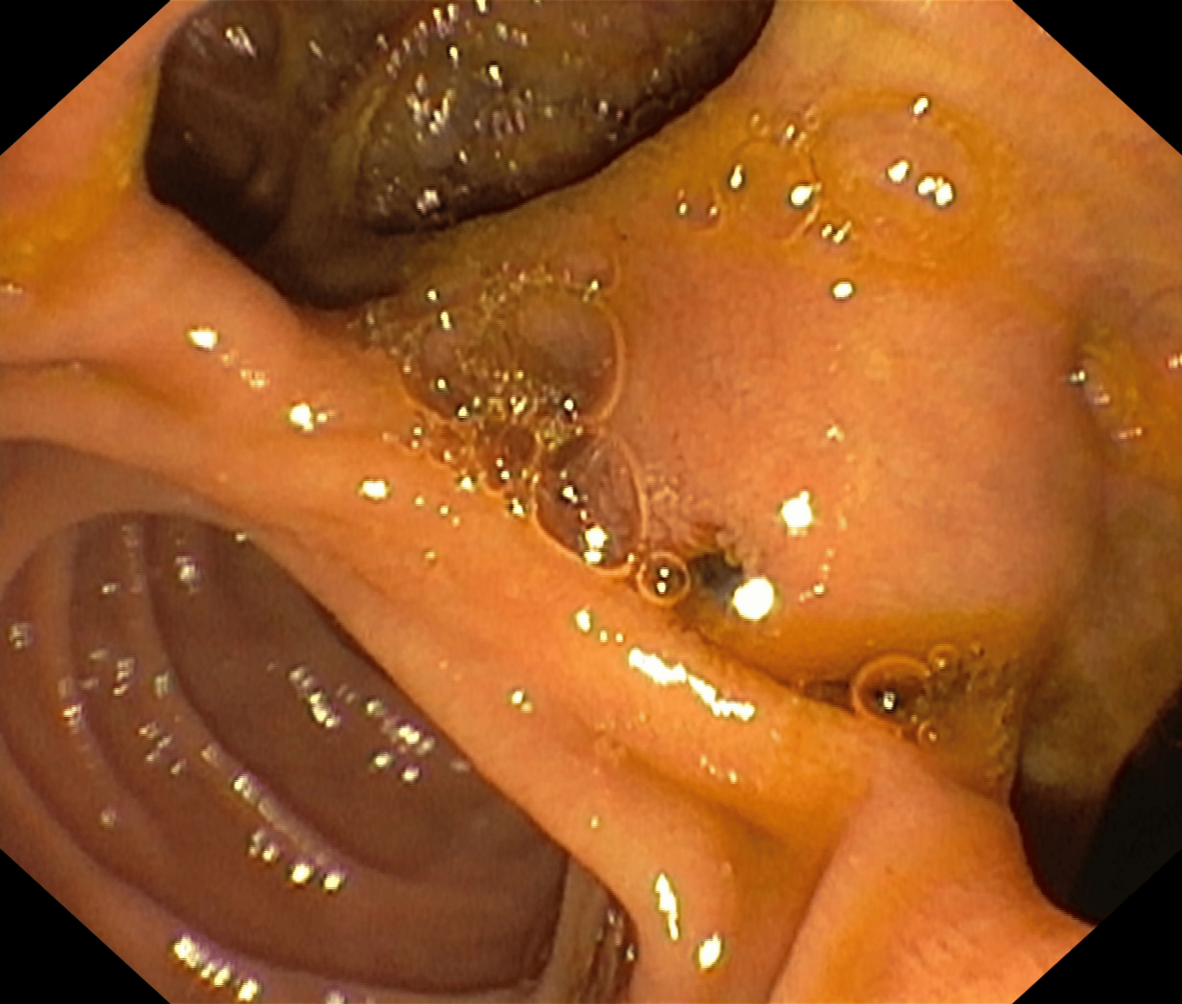
Papilla of Vater situated in a large diverticular space with a stone discretely protruding through the orifice.

The morphological structure of the pancreas was not impaired. We decided first to perform a duodenoscopy with endoscopic treatment of the choledocholithiasis and then to refer the patient to the surgery department. During endoscopy, we found the papilla of Vater situated in a large diverticular space with a slightly bulging appearance. Discretely protruding through the orifice, we observed the stone (Figure 7).

We conducted endoscopic retrograde cholangiopancreatography and guidewire-assisted papilosphincterotomy, followed by basket extraction of several mixed stones from the common bile duct (Video 1).

**Video 1:** Extraction of several mixed stones from the common bile duct.

After the endoscopic procedure, there were no complications. The liver enzymes normalized within a few days. The patient was discharged and referred to a surgeon for laparoscopic deconnection of the biliodigestive fistula and cholecystectomy. During follow-up after six and 12 months, the patient presented asymptomatic with no laboratory or imaging abnormalities.

## Discussion

Gallstone disease is a common gastrointestinal problem. Plenty of factors lead to gallstone formation, including gender, genetics, obesity, rapid weight loss, diabetes, hypertriglyceridemia, drugs, total parenteral nutrition, infections, liver cirrhosis, hemolysis, ileal diseases, and pregnancy [5]. Abnormal metabolism of cholesterol, bile acids, bilirubin, and lecithin leads to supersaturation, nucleation, microstone and gallstone formation. There are various gallstone colors, shapes, and sizes [6]. Cholecystoenteric fistulas are a rare complication of chronic gallstone disease and occur in 0.1-0.9% of patients with choledocholithiasis, more often diagnosed in older females with comorbidities [1]. Cholecystoenteric fistulas were first described by Thomas Bartholin in 1654 [7]. Chronic cholecystitis leads to adhesions to the surrounding tissues. The gallstones cause pressure and erosions on the nearby tissues with chronic inflammation, necrosis, and finally fistula formation [5]. Spontaneous tracts between the gallbladder and the digestive tract might also be caused by peptic ulcers, biliopancreatic junction or pancreatic neoplastic infiltration, Caroli’s disease, liver or renal abscesses, penetrating trauma or necrotic colitis, inflammatory bowel diseases, diverticulitis or previous surgery [2,5]. The altered physiology, which lacks protective mechanisms, can cause bile reflux into the duodenum or enteric content into the biliary tract [3].

Cholecystoduodenal fistula is the most common type of cholecystoenteric fistulas, found in 60-85% of patients, followed by cholecystocolic and cholecystogastric fistulas [4,8]. Patients are asymptomatic for a long period. They can report a nonspecific vast array of symptomatology including abdominal pain, fever, bloating, nausea, vomiting, diarrhea, malabsorption, weight loss, and jaundice [5,9]. The complications of untreated cholecystoenteric fistulas are even rarer, which can lead to a delay in diagnosis [1,5]. Gallstone ileus is caused by the passage of one or more gallstones through the fistula into the bowel to the terminal ileum with increasing volume during the passage leading to ileal obstruction, gastric outlet obstruction (Bouveret syndrome) secondary to impaction of a gallstone in the proximal duodenum with or without acute pancreatitis, bleeding, suppurative cholangitis or liver abscess possibly by the enteric content backflow into the biliary tract [1,5]. The diagnosis of cholecystoenteric fistulas is rarely the first suspicion and is often diagnosed late due to nonspecific symptoms or complications [1-5]. Only 8%-17% of bilioenteric fistulas are diagnosed on preoperative imaging [9]. Laboratory tests are usually nonspecific and depend on the severity of inflammation; however, we can observe hyperbilirubinemia, liver enzyme increase, leukocytosis, electrolyte abnormalities, and renal failure [6]. At abdominal ultrasound examination, the presence of pneumobilia may be the key feature. For the proper diagnosis of cholecystoenteric fistulas and their complications, we can use computed tomography, magnetic resonance, or endoscopic retrograde cholangiopancreatography [9,10]. Computed tomography is the imaging technique of choice as it is widely available with high specificity and sensitivity above 90% and can directly show pathological communication, intestinal obstruction, and aerobilia [1,9-12]. Magnetic resonance cholangiopancreatography provides better information on the anatomy of the biliary tree, delineates fluid from calculi, demonstrates isoattenuating stones, and is useful in patients who cannot tolerate the contrast medium [13]. Patients with biliodigestive fistulas are treated successfully by laparoscopic surgery. Robotic surgery nowadays is becoming a more desired technique as it gives excellent visualization, dexterity, control, and maneuverability [9].

In our presented case, the age and gender of the patient are not the common circumstances for biliodigestive fistula. Moreover, he was admitted with unspecific complaints and liver dysfunction with leading cholestasis with increased alkaline phosphatase and GGT. The quality of the ultrasound examination and the presence of chronic cholecystitis with aerobilia gave us the suspicion of biliodigestive fistula. At computed tomography, we observed a gallstone in the common bile duct above the papilla of Vater, causing dilation and liver dysfunction. The stone did not block the whole flow of bile through the common bile duct at that time, therefore the bilirubin levels were in the reference range. As we already suspected pneumobilia and excluded other causes such as a biliary-enteric surgical anastomosis and an incompetent sphincter of Oddi, we were able to diagnose cholecystoduodenal fistula using computed tomography, which was early enough, before any severe complications occur. We discussed the case at a multidisciplinary meeting and decided first to perform endoscopic extraction of the gallstone and then to refer the patient for laparoscopic surgery. During endoscopy, we found a large periampullary duodenal diverticulum with the papilla orifice located in the diverticulum, classified as type IIIA according to a novel classification system, developed by expert endoscopists and published in 2021 [11]. Periampullary duodenal diverticula are found in 5-33%, as a recent Swedish retrospective cohort study reported an incidence of 10.2% during endoscopic retrograde cholangiopancreatography [12]. They are associated more often with choledocholithiasis and cholangitis. The periampullary duodenal diverticulum additionally challenged the required papillosphincterotomy. It is not unusual to miss a diverticulum at computed tomography; however, periampullary duodenal diverticulum leads to less successful cannulation, which can increase the risk of complications of invasive endoscopic procedures such as perforation [12]. We were able to perform successful endoscopic retrograde cholangiopancreatography, papillosphincterotomy, and removal of several stones from the common bile duct. After the endoscopic procedure, there were no complications. The liver enzymes normalized within a few days and the size of the common bile duct normalized. Three weeks later, the patient underwent a laparoscopic deconnection of the biliodigestive fistula and cholecystectomy. We followed up with the patient after six and 12 months. He presented asymptomatic and in excellent overall condition with no laboratory or imaging abnormalities.

## Conclusions

Cholecystoenteric fistulas are a rare entity and challenging disease diagnostic and therapeutic process. Patients are usually with no specific symptoms. If not diagnosed on time, cholecystoenteric fistulas can lead to life-threatening conditions. Cholecystoenteric fistulas should be part of the differential diagnosis of aerobilia and liver dysfunction as computed tomography is an available helpful imaging technique. Other complications of chronic cholecystitis such as choledocholithiasis might be also present and additionally challenge the treatment of the patients. When there is a high clinical suspicion, we need early, dedicated, and proper diagnostic imaging and endoscopic techniques to be further able to individualize the management strategies based on the patient's condition, including surgical, endoscopic, robotic, or conservative approaches.
